# Safety and Effectiveness of Triclosan-Coated Polydioxanone (PDS Plus) versus Uncoated Polydioxanone (PDS II) Sutures for Prevention of Surgical Site Infection after Hypospadias Repair in Children: A 10-Year Single Center Experience with 550 Hypospadias

**DOI:** 10.3390/biomedicines12030583

**Published:** 2024-03-06

**Authors:** Zenon Pogorelić, Lana Stričević, Sara Elezović Baloević, Jakov Todorić, Dražen Budimir

**Affiliations:** 1 Department of Pediatric Surgery, University Hospital of Split, 21000 Split, Croatia; 2 Department of Surgery, School of Medicine, University of Split, 21000 Split, Croatia

**Keywords:** hypospadias, surgical site infection, SSI, hypospadias repair, complications, triclosan, triclosan-coated polydioxanone, PDS Plus

## Abstract

Aim: Triclosan is an antiseptic substance that has been shown in preclinical studies to reduce bacterial load in the wound and slow bacterial growth by inhibiting fatty acid synthesis. It is claimed that the coating protects against colonization of the tissue around the suture. This study aimed to compare the safety and efficacy of triclosan-coated polydioxanone versus uncoated polydioxanone sutures for the prevention of surgical site infections (SSIs) following hypospadias repair in children. Methods: The medical records of 550 children who underwent hypospadias repair between 1 January 2014 and 31 December 2023 were retrospectively analyzed. The patients included in the study were divided into two groups. The first group consisted of the patients in whom polydioxanone (PDS II) was used (*n* = 262), while in the patients of the second group (*n* = 288), triclosan-coated polydioxanone (PDS Plus) was used for hypospadias repair. Secondary outcomes were defined as the occurrence of early and late complications, the number of readmissions within 30 days after surgery (ReAd), unplanned return to the operating room (uROR), and repeat operations. Results: The median age of all children enrolled in the study was 16 (IQR 14, 20) months. The patients in whom PDS Plus was used for hypospadias repair had a significantly lower number of SSIs than the patients in whom PDS II was used (*n* = 18 (6.9%) vs. *n* = 4 (1.4%), *p* < 0.001). Wound infection led to wound dehiscence in 10 of 18 patients from the PDS II group, while all four wound infections from the PDS Plus group led to wound dehiscence (*p* = 0.07). The number of postoperative urethrocutaneous fistulas was significantly lower in the patients in whom PDS Plus was used (13.7% vs. 8.3%, *p* = 0.042). The incidence of late complications did not differ between the study groups: meatal stenosis (*p* = 0.944), residual chordee (*p* = 0.107), urethral stricture (*p* = 0.196), scarring (*p* = 0.351) and urinary discomfort (*p* = 0.713). There were no cases of uROR in either group. The ReAd rate was low in both groups (*n* = 5 (1.9%) vs. *n* = 2 (0.6%), *p* = 0.266). The frequency of reoperations was lower in the group of patients treated with PDS Plus than in the group of patients treated with PDS II (11.1% vs. 20.6%; *p* = 0.03). Conclusion: The use of PDS Plus in hypospadias surgery significantly reduces the incidence of SSI, postoperative fistulas, and reoperation rates compared to PDS II.

## 1. Introduction

Hypospadias is a very common congenital anomaly of the male genitalia, with an incidence of 1/250 newborns [1]. The meatus of the urethra is not in its normal position but is displaced along the ventral side of the penis. This anomaly results from incomplete virilization of the genital tubercle, which leads to incomplete closure of the glans and penile structures during embryogenesis [2]. The main features of hypospadias are an ectopic position of the urethral meatus along the ventral side of the penis, anywhere from the glans to the perineum, a ventral curvature of the penis and a ventral defect of the foreskin [3]. There are special forms of hypospadias for which the term hypospadias sine hypospadias is used because the meatus of the urethra is in its normal position but there is a ventral curvature of the penis and a distorted foreskin. The other special form of hypospadias is megameatus intact prepuce, which is characterized by a recumbent meatus next to an unclosed glans with a very wide open navicular fossa and a normally receded round foreskin. Depending on the preoperative position of the meatus, hypospadias are often divided into anterior, penile and posterior. In about two thirds of hypospadias, the urethral meatus is distal to the penile shaft and is considered a mild form that is usually not associated with other urogenital deformities. The remaining third of hypospadias is proximal and is often more severe.

The exact etiology of the hypospadias is not known with certainty, but there is much evidence to suggest the influence of hormones during embryogenesis and a hereditary predisposition [2,4]. The surgical approach is the only treatment modality. The goal of almost every surgical technique used in the treatment of hypospadias is to achieve both cosmetic and functional normality and to increase the child’s self confidence [2,3,4]. Different surgeons have different preferences as to when the optimal time for surgical correction of this anomaly is, but most agree that it is between the sixth and eighteenth month of life. This consensus has been reached by attempting to balance factors such as age-related genital dimensions and anesthetic risks, as well as the effects of toilet training and the potential psychological consequences of delaying surgery until an age when the child is genitally aware and remembers the procedure [5,6,7,8,9,10,11].

Today, there are more than 250 different techniques to correct this anomaly, which shows that there is no ideal technique [1,8,11]. The reported complication rate after hypospadias correction is between 5% and 70% [1,7]. The complications are usually due to several interrelated factors. These include factors related to the severity of the hypospadias and the characteristics of the urethral plate, factors related to the patient (e.g., age at the time of surgery, endocrine environment, and wound healing disorders), and finally factors related to the surgeon (e.g., surgeon’s experience, choice of technique and postoperative management) [7]. Acute complications occur in the majority of cases within 10 days of hypospadias repair and require appropriate assessment and decision making for management [4,7,9]. Major complications associated with failed hypospadias repair include residual curvature, healing complications (surgical site infections—SSIs leading to wound dehiscence, urethrocutaneous fistula formation and urethral breakdown), urethral obstruction (meatus stenosis, urethral stricture, and functional obstruction), urethral diverticulum, hairy urethra, and penile skin deficits [3,7].

The complications can be divided into acute complications such as fistula, wound infection, wound dehiscence, skin necrosis; and late complications such as meatus stenosis, residual chordee, urethral stricture, scar, and difficulty urinating. The development of urethrocutaneous fistulas (UCFs) is still the most important postoperative complication of repaired hypospadias and is between 12% and 90%. Healing occurs spontaneously in about 30% of patients, provided there is no distal obstruction [8,9,10,11]. In general, redo surgery for a complication is usually performed six months after the initial hypospadias repair, unless immediate exploration is indicated by bleeding, infection, or debridement [11].

Among pediatric urologists who treat children with hypospadias, there is no general consensus on the choice of suture material. Sutures may be one of the causes of early and late complications in children undergoing surgery for this anomaly. There is consensus that the suture material used for hypospadias repair should be absorbable. At the same time, it is of great importance that the suture material has sufficient mechanical strength to support the wounds during the healing process. Non-absorbable suture material is not used in hypospadias surgery due to the risk of stone formation if it comes into contact with urine. There are several studies that have investigated the effects of suture technique and suture material on the complication rate. According to the conclusions of these studies, the use of polydioxanone (PDS) can significantly reduce the complication rate after hypospadias repair. In addition, another study supports the use of PDS over polyglactin in urethroplasty [12]. Tovar et al. have compared SSIs after abdominal fascial closure with triclosan-coated suture versus PDS loop suture, and they proved that the use of triclosan-coated suture reduces the incidence of wound infection. Markus et al. have compared the efficacy of triclosan-coated PDS Plus with uncoated PDS II and found that coated sutures reduce the incidence of SSIs by only 1.3% [13]. 

Triclosan [2,4,4-trichloro-2-hydroxydiphenyl ether] is an antimicrobial agent that has been shown to reduce bacterial load in a wound and slow bacterial growth by inhibiting fatty acid synthesis. At standard concentrations, triclosan has no unwanted side effects. It is claimed that the coating provides protection against bacterial colonization of the tissue around the suture for almost 30 days and prevents the formation of a ligature abscess. In vitro studies have shown that triclosan forms a zone of inhibition around the suture material and is effective against the most common pathogens of SSIs, particularly Gram-positive bacteria [14,15].

The aim of our study is to compare the safety and efficacy of triclosan-coated PDS Plus sutures versus uncoated PDS II sutures for the prevention of SSIs after hypospadias repair in children.

## 2. Materials and Methods

### 2.1. Patients

**Figure 1:**
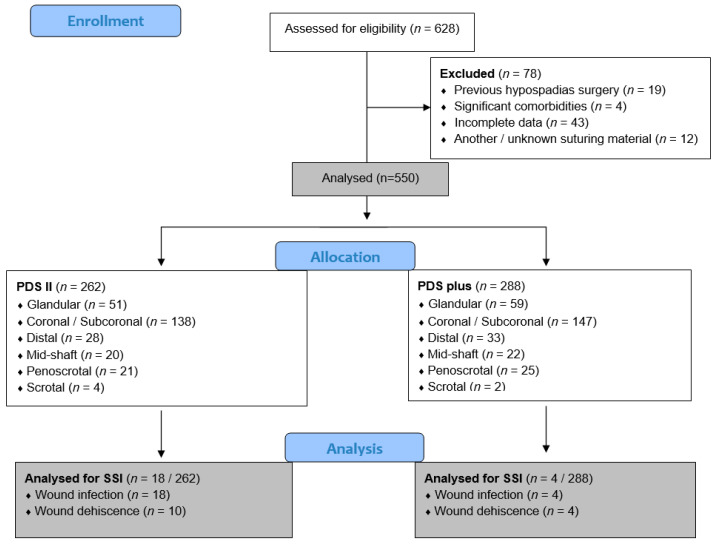
Flowchart of the study. Legend: PDS—Polydioxanone; SSI—surgical site infection.

A retrospective analysis of the case files of 628 children who underwent hypospadias repair at the Department of Pediatric Surgery of the University Hospital of Split between 1 January 2014 and 31 December 2023 was performed. As they did not meet the criteria for inclusion in the study, a total of 78 children were excluded from further analysis. Finally, 550 children met the inclusion criteria and were further analyzed. A flowchart of the study is shown in Figure 1.

The Inclusion criteria were pediatric patients (aged 0 to 17 years) operated on for hypospadias at our institution by three experienced pediatric urologists (D.B., J.T., and Z.P.) using polydioxanone (PDS II^®^ 6/0, Ethicon, Johnson & Johnson, Diegem, Belgium) or triclosan-coated polydioxanone (PDS Plus^®^ 6/0, Ethicon, Johnson & Johnson, Diegem, Belgium) sutures. The patients outside the indicated age range, the patients who had been operated on previously for hypospadias, the patients in whom a suture material other than PDS was used, the patients who were operated on by a surgeon other than those mentioned above, the patients with significant comorbidities that may affect wound healing, such as immunodeficiency or diabetes mellitus, the patients with less than 30 days of follow-up, or those with incomplete data in case files were excluded from the further analysis. 

### 2.2. Ethical Aspects

The study met the ethical standards of the institutional and national research committee and the 1964 Declaration of Helsinki and its subsequent amendments or comparable ethical standards, and the Institutional Review Board of University Hospital of Split approved the study (approval number: 500-03/23-01/222; date of approval: 27 November 2023).

### 2.3. Outcomes of the Study

The main outcome was the incidence of SSIs in children operated on with triclosan-coated and uncoated polydioxanone sutures. The secondary outcomes of the study were the frequency of other complications, the number of readmissions within 30 days of surgery (ReAd) [16], unplanned returns to the operating room (uROR) [17], and the rate of repeat surgery between groups.

### 2.4. Data Collection and Study Design

Depending on the suture used for hypospadias repair, the patients who met the inclusion criteria and were included in the analysis were divided into two study groups. The first group consisted of the patients in whom polydioxanone (PDS II^®^) was used, while triclosan-coated polydioxanone (PDS Plus^®^) was used for hypospadias repair in the patients of the second group. The groups were compared with respect to patient baseline demographics (age, weight, and height), type of hypospadias (glandular, subglandular, coronal, subroroneal, distal, mid-shaft, penoscrotal, or scrotal), and preoperative urinary difficulties (meatal stenosis, dripping or straining). In addition, the groups were compared in terms of early (fistula, wound infection, wound dehiscence, and skin necrosis) and late complications (meatal stenosis, residual chordee, urethral stricture, scar, urinary tract symptoms), repeat operations, ReAd, uROR, and microbiologic isolates.

### 2.5. Surgical Techniques and Suturing Material

The following surgical techniques have been used for repair of glandular, subglandular, coronal, and subcoronal hypospadias: Mathieu, the meatal advancement and glanduloplasty (MAGPI), Snodgrass, and the meatal mobilization (MEMO) [18,19,20,21]. For distal or middle hypospadias, the techniques of Koff or Snongrass were used [22]. For the repair of scrotal and penoscrotal hypospadias, two-stage tubularized urethroplasty was performed using buccal mucosa or free foreskin grafts [23]. The surgical technique was based on the surgeon’s decision and preferences. From 1 January 2014 to 31 December 2018, polydioxanone was used as the suture material, whereas from 1 January 2019 to 31 December 2023, triclosan-coated polydioxanone was used as the suture material.

### 2.6. Postoperative Protocol and Follow-Up

At the end of the procedure, 1% lidocaine (Lidokain, Belupo, Koprivnica, Croatia) was applied subcutaneously. The standard wound dressing consisted of vaseline gauze, silver sulfadiazine cream (Dermazin, Salutas Pharma GmbH, Osterweddingen, Germany), and COBAN™ self-adherent wrap (3M™, Neuss, Germany). The dressing was changed on postoperative days 3 and 7, or more frequently if the local status required it. Paracetamol (Paracetamol Kabi, Fresenius Kabi, Zagreb, Croatia) at a dose of 10–15 mg/kg or ibuprofen (Brufen, Mylan, Zagreb, Croatia) at a dose of 10 mg/kg were administered for pain relief. In most cases, cephalexin (Cefalexin, Belupo, Koprivnica, Croatia) at a dose of 25–50 mg/kg or gentamicin (Gentamicin, Belupo, Koprivnica, Croatia) at a dose of 3–5 mg/kg were used for antibiotic prophylaxis until the removal of the urinary catheter. The urinary catheter was removed 7 to 14 days after surgery, depending on the type of hypospadias and the surgeon’s preference. After discharge, patients were followed up in our outpatient clinic on the 7th to 14th day after surgery, depending on the type of hypospadias and the surgeon’s preference, in order to check the wound and detect any complications. The follow-up program consisted of a physical examination and, if necessary, dilatation of the urethra one, three, six, and twelve months after the operation to assess the presence of late complications. If the patient’s condition required it, visits were scheduled more frequently than usual.

### 2.7. Statistical Analysis

Statistical analysis of the data was performed using the Statistical Package for the Social Sciences—SPSS 28.0 (IBM Corp, Armonk, NY, USA) and Microsoft Excel for Windows Version 11.0 (Microsoft Corporation, Redmond, WA, USA). The distribution of quantitative data was described by median and interquartile range (IQR). Absolute numbers and percentages were used to describe numerical variables. The Mann–Whitney U test was used to compare continuous variables, while the Chi-square test was used to compare categorical variables. A two-tailed Fisher’s exact test was used when the frequency of events in a given cell was low. All *p*-values of less than 0.05 were considered significant.

## 3. Results

### 3.1. Preoperative Characteristics of the Patients

The median age of all children included in this study was 16 (IQR 14, 20) months. The majority of patients in both groups had glandular, coronal, or subcoronal hypospadias (PDS II–72.7% vs. PDS Plus–71.5%), while the incidence of distal/middle shaft hypospadias in the PDS II and PDS Plus groups was 18.3% and 18.1%, respectively. The lowest incidence was found for the penoscrotal/scrotal type of hypospadias (PDS II–9.5% vs. PDS Plus–9.4%). The incidence of preoperative urinary difficulties was very low in both groups. Both groups were symmetrical in terms of demographic characteristics, type of hypospadias, and preoperative urinary difficulties. Patient demographic and preoperative clinical data are shown in Table 1.

### 3.2. Analysis of the Outcomes of the Study

**Figure 2:**
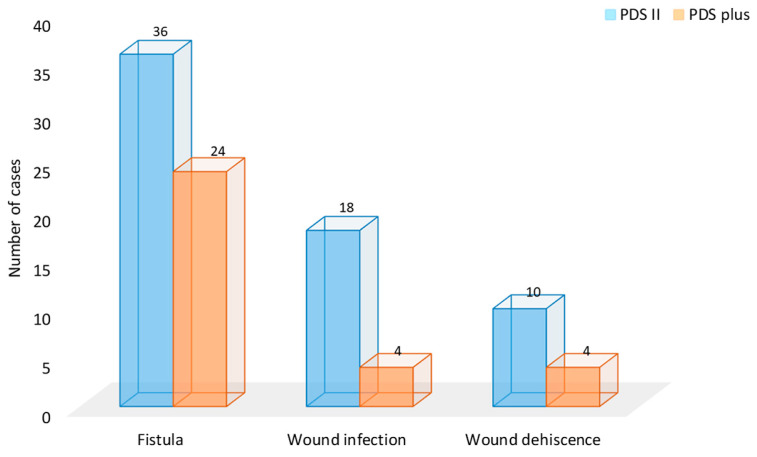
Comparison of early complications between the study groups.

Regarding the main outcome of the study—SSI rate—the patients in whom the triclosan-coated polydioxanone suture was used for hypospadias repair had a significantly lower number of SSIs than the patients in whom uncoated polydioxanone was used (*n* = 18 (6.9%) vs. *n* = 4 (1.4%), *p* < 0.001). Wound infection led to wound dehiscence in 10 of 18 patients from the PDS II group, whereas all four wound infections from the PDS Plus group led to wound dehiscence (*p* = 0.07). All children with a dehiscence of the surgical wound were initially treated with a normal local wound dressing. After healing of the infection, the wound was healed secondarily and a repeat surgery was performed approximately four to six months after the first operation, depending on the local status and preferences of the operating surgeon. The number of postoperative fistulas was significantly lower in patients in whom triclosan-coated polydioxanone was used (*p* = 0.042) (Figure 2). 

The incidence of late complications did not differ between study groups: meatus stenosis (*p* = 0.944); residual chordee (*p* = 0.107); urethral stricture (*p* = 0.196); scarring (*p* = 0.351); and urinary difficulties (*p* = 0.713). There were no cases of uROR in either group. The rate of ReAd was low in both groups (*n* = 5 (1.9%) vs. *n* = 2 (0.6%), *p* = 0.266). The incidence of reoperation was lower in the group of patients using triclosan-coated polydioxanone compared with those treated with uncoated polydioxanone (11.1% vs. 20.6%; *p* = 0.03). A comparison of complications between the study groups’ ReAd and redo surgery rates is shown in Table 2.

A decrease in the incidence of wound infections was noted in all hypospadias types treated with triclosan-coated polydioxanone compared with those treated with uncoated polydioxanone, with statistical significance noted in the most common types—coronal and subcoronal hypospadias (*p* = 0.030). A detailed analysis of wound infections according to the type of hypospadias is presented in Table 3.

The most common cause of wound infections in both groups was *Staphylococcus aureus*, followed by *Enterobacteriaceae* and *Pseudomonas aeruginosa*. The microbiological cultures of wounds exudate in children with SSIs is demonstrated in Table 4. 

## 4. Discussion

Since surgical site infections are an important complication that can impair wound healing and prolong hospitalization, various measures are taken to prevent them, such as antibiotic prophylaxis, preoperative skin cleansing, and the use of suture material coated with antibiotic substances. This study compared triclosan-coated (PDS Plus) and uncoated polydioxanone sutures (PDS II) in 550 patients undergoing hypospadias repair surgery. The study found a lower number of SSIs in patients in whom PDS Plus suture was used, with statistical significance for the most common type (coronal/subcoronal). The number of postoperative fistulas, the most common complication, was also lower in this group. The number of late complications (meatal stenosis, residual chordee, urethral stricture, scarring, voiding dysfunction), on the other hand, did not differ between the groups. The ReAd rates were low in both groups, and the rate of repeat procedures was lower in patients with PDS Plus sutures. The most common microbiological isolates were *Staphylococcus aureus* and *Enterobacteriaceae*, which is consistent with previously published data [3].

For hypospadia repair, surgery absorbable sutures should be used. As there is still no definitive agreement on the suture material, many studies have attempted to compare the success and complications of surgery with different suture materials. In a study by Mohamed Ali Alaraby, which showed a lower rate of postoperative complications (34% vs. 10.9%) and better results when using PDS for hypospadias repair, polydioxanone proved to be better than Vicryl [11]. Another study compared PDS and Vicryl in hypospadias repair and showed that complications occurred more frequently with Vicryl sutures than with PDS sutures (15.1% vs. 5.3%). In addition, it was found that the interval between surgery and the occurrence of complications was shorter in the Vicryl group [24].

PDS Plus is a triclosan-coated suture material, an antiseptic agent that prevents bacterial colonization of the suture material and the surrounding tissue and is particularly effective against Gram-positive bacteria. Our results supported the use of PDS Plus as a suture material in the prevention of SSIs, which is consistent with several other studies comparing PDS and PDS Plus suture material. A randomized, controlled clinical trial by Ruiz-Tovar et al. [15] compared triclosan-coated and uncoated polydioxanone sutures in emergency abdominal procedures and found a statistically significant lower rate of SSIs when coated sutures were used. A prospective observational study in Spain, which included five surgical specialties, found a statistically significant 36% reduction in SSIs when triclosan-coated sutures were used [25]. A meta-analysis comparing triclosan-coated and uncoated sutures (Vicryl, Monocryl, and PDS), which included seventeen randomized control trials (RCT), showed a significant 30% reduction in the SSI rate [26]. A few other meta-analyses also confirm a lower risk of SSIs when using triclosan-coated sutures. Ahmed I et al. included twenty-five RCTs in their meta-analysis and reported a significantly lower rate of SSIs with the use of coated sutures, in both adult and pediatric patients, in clean and contaminated wounds [27]. Similar results were found by Daoud et al. [28] and Edwards et al. [29], who have reported a statistically significant lower risk of SSIs with the use of coated sutures compared to uncoated.

In contrast, another meta-analysis comparing triclosan-coated and uncoated PDS in hip and knee arthroplasty found no significant difference in the reduction in the incidence of superficial and deep SSIs between the two groups [30]. An RCT comparing Vicryl and Vicryl Plus in primary hip and knee arthroplasty found no significant difference in SSIs between these two groups [31]. Another study comparing Vicryl and Vicryl Plus in leg wound infections in patients undergoing coronary artery bypass grafting showed no advantage in using coated suture compared to uncoated, as the infection rate was almost the same in both groups (10%) [32].

A study by Baracs et al. compared SSIs after abdominal wall closure in colorectal surgery [14]. In their study, no significant differences in SSIs were found in the two groups, as the SSI rate was almost the same in percentage terms (10%). Another multicenter, randomized, controlled trial showed no difference in the prevention of SSIs when using PDS Plus sutures compared to PDS II. There was also no difference in secondary outcomes such as wound dehiscence, length of postoperative hospital stay, and quality of life [13]. A meta-analysis by Sandini et al. included six studies comparing triclosan-coated and uncoated sutures in abdominal wall closure [33]. The single-center studies showed a significant reduction in SSIs when coated sutures were used, in contrast to multicenter studies in which such a reduction could not be demonstrated. Similar results were found by Onesti et al. Of fifteen RCTs analyzed, seven showed a significant advantage of using coated sutures, while eight showed no difference [34].

The reason for these contradictory results in the different studies could be due to different study types, different sample sizes, statistical methods, definitions of SSI, risk factors for SSIs such as diabetes or immunodeficiency, operation time, different experiences of the surgeon, and different lengths of follow-up. The different types of procedures included in some of these studies may also contribute to inconsistent results. All this suggests that there is a need for studies comparing the incidence of SSIs in the same type of procedures with clear inclusion and exclusion criteria, experienced surgeons, and a large number of patients.

Several other factors can influence infections at the surgical site. Systemic factors such as concomitant diseases, nutritional deficiencies, therapeutic agents, and age can influence the occurrence of SSIs. In addition, local factors such as blood supply, wound tension, or surgical technique are also very important for the prevention of SSIs. The appropriate use of surgical gloves is crucial to prevent the transmission of infections from the medical staff to the patient. Regardless of the type of material the gloves are made of, mini-perforations on gloves are a common occurrence and can be a source of microorganism transmission during surgical procedures. The recent literature suggests that the perforation rate of gloves can be as high as 30%. Recent studies recommend changing surgical gloves after two hours if the procedure lasts longer than two hours [35]. In addition, prolonged hospitalization has been identified as a potentially modifiable factor for SSIs in general surgery patients [36]. It is advisable to discharge the patient as soon as possible. This is one reason why we have in recent years discharged patients within 1 to 2 days after surgery with urinary catheters.

There are several limitations of our study. First, it is a retrospective study, which means that some information about the patients that might be important for the results is not available. Second, the fact that this is a single-center study implies that the results may not be generalizable to the general population. Finally, although the sample is large enough, it is not randomized, so it is susceptible to selection bias and is not representative of the general population. In view of the contradictory results of the studies available today, further randomized, prospective and multicentric studies with large samples should be conducted and, finally, a systematic review and meta-analysis should be carried out.

## 5. Conclusions

The present study demonstrates the benefit of using triclosan-coated PDS sutures in hypospadias surgery by reducing the incidence of SSI. In addition, a reduction in the incidence of urethrocutaneous fistulas, the most common complication of hypospadias surgery, was observed with the use of triclosan-coated PDS sutures. However, SSIs remain an unresolved problem in hypospadias surgery, and as the currently available studies show contrary results, further prospective, randomized studies are needed to prove the benefit of coated sutures.

## Figures and Tables

**Table 1:** Comparison of demographic characteristics of patients and types of hypospadias between the study groups.

Variables	Group I (*n* = 262)	Group II (*n* = 288)	*p*
	PDS II	PDS Plus	
Demographic characteristics of patients; median (IQR) or *n* (%)			
Age (months)	17 (13, 22)	14 (12, 20)	0.015 *
0–12	66 (25.2)	74 (25.7)	0.260 ^†^
12–24	138 (52.7)	169 (58.7)	
24–48	41 (15.6)	32 (11.1)	
>48	17 (6.5)	13 (4.5)	
Weight (kg)	9.2 (7.9, 10.4)	8.9 (7.5, 10.1)	0.235 *
Height (cm)	76.7 (68.5, 86)	76.1 (66, 85)	0.729 *
Type of hypospadias; *n* (%)			
Glandular	51 (19.5)	59 (20.5)	0.994 ^†^
Coronal/Subcoronal	138 (52.7)	147 (51)	
Distal	28 (10.7)	33 (11.5)	
Mid-shaft	20 (7.6)	22 (7.6)	
Penoscrotal/Scrotal	25 (9.5)	27 (9.4)	
Preoperative urinary difficulties; *n* (%)			
Meatal stenosis	3 (1.1)	2 (0.6)	0.672 ^‡^
Dripping	2 (0.7)	1 (0.3)	0.607 ^‡^
Straining	1 (0.4)	1 (0.3)	1.000 ^‡^

* Mann–Whitney U-test. ^†^ Chi-square test. ^‡^ Fisher’s exact test. PDS—polydioxanone; IQR—interquartile range.

**Table 2:** Comparison of complications: ReAd and redo surgery rates between the study groups.

Variables	Group I (*n* = 262)	Group II (*n* = 288)	*p*
	PDS II	PDS Plus	
Early complications; *n* (%)			
Urethrocutaneous fistula	36 (13.7)	24 (8.3)	0.042 *
Wound infection	18 (6.9)	4 (1.4)	0.001 *
Wound dehiscence	10 (3.8)	4 (1.4)	0.070 *
Skin necrosis	1 (0.4)	1 (0.3)	1.000 ^†^
Late complications; *n* (%)			
Meatal stenosis	16 (6.1)	18 (6.2)	0.944 *
Residual chordee	3 (1.1)	0 (0)	0.107 ^†^
Urethral stricture	4 (1.5)	1 (0.3)	0.196 ^†^
Scarring	3 (1.1)	1 (0.3)	0.351 ^†^
Urinary difficulties	4 (1.5)	3 (1)	0.713 ^†^
ReAd/Redo surgery; *n* (%) or Median (IQR)			
ReAd	5 (1.9)	2 (0.6)	0.266 ^†^
Redo surgery	54 (20.6)	32 (11.1)	0.003 *
Day of SSI	5 (3, 6)	4 (3, 6)	0.547 ^‡^

* Chi-square test. ^†^ Fisher’s exact test. ^‡^ Mann–Whitney U-test. PDS—polydioxanone; ReAd—readmission within 30 days after index surgery; SSI—surgical site infection; IQR—interquartile range.

**Table 3:** Comparison of infection rates according to type of hypospadias between the study groups.

Variables *n (%)*	Group I (*n* = 262)	Group II (*n* = 288)	*p* *
	PDS II	PDS Plus	
Glandular	3/51 (5.9)	0/59 (0)	0.096
Coronal/Subcoronal	9/138 (6.5)	2/147 (1.4)	0.030
Distal	2/28 (7.1)	1/33 (3)	0.589
Mid–shaft	2/20 (10)	0/22 (0)	0.220
Penoscrotal/Scrotal	2/25 (8)	1/27 (3.8)	0.602

* Fisher’s exact test; PDS—polydioxanone.

**Table 4:** Microbiological cultures of wounds in children with surgical site infection.

Microbiological Isolate *n (%)*	Group I (*n* = 15) *	Group II (*n* = 4)
	PDS II	PDS Plus
*Staphylococcus aureus*	7 (46.7)	2 (50)
*Pseudomonas aeruginosa*	2 (13.3)	1 (25)
*Streptococcus pyogenes*	1 (6.7)	0 (0)
*Escherichia coli*	2 (13.3)	1 (25)
*Enterococcus faecalis*	2 (13.3)	0 (0)
*Klebsiella pneumoniae*	1 (6.7)	0 (0)

* No microbiological findings were available in three patients from group I.

## Data Availability

The data supporting this study’s findings are available upon request from the corresponding author.
